# Supplementary material for: Airborne influenza virus shedding by patients in health care units: Removal mechanisms affecting virus transmission
Source: PLoS One. 2023 Oct 25;18(10):e0290124. doi: 10.1371/journal.pone.0290124 (PMC10599543; doi:10.1371/journal.pone.0290124)
Supplement: S1 File — (DOCX) [file pone.0290124.s001.docx]

**Supporting Information**

Table S1: Model input parameters and assumptions

| Parameter | Unit | Definition | Value/range used | Reference / Justification |
| --- | --- | --- | --- | --- |
| u_x_ | m/sec | Coughing speed | 2.2 - 22 | [1-3] |
| u_y_, u_z_ | m/sec | Lateral velocities | 0.125 – 0.250 | [4] |
| M | RNA copies/cough | Shedding rate | p-norm (mean = 15.8; sd = 29.3) | [5] |
| σ | N.m-1 | Surface Tension | 0.072 | [6] |
| M_w_ | g.mol^-1^ | Molar mass of water | 18 | - |
| M_NaCl_ | g.mol^-1^ | Molar mass of NaCl | 58.4 | [7] |
| M_TP_ | g.mol^-1^ | Molar mass of total protein | 66.5 x 10^3^ | [7] |
| $\rho_{NaCl}$ | g.m^-3^ | Density of NaCl | 0.997 | [7] |
| $\rho_{TP}$ | g.m^-3^ | Density of Total protein | 2.165 | [7] |
| $\rho_{w}$ | g.m^-3^ | Density of water | 1.362 | - |
| R | J/k.mol | Ideal Gas constant | 8.31 | - |
| v_NaCl_ | - | Stoichiometric dissociation number of NaCl | 2 | [6] |
| v_TP_ | - | Stoichiometric dissociation number of TP | 1 | [6] |
| x_NaCl_ | % | NaCl mass fraction of droplet | 0.104 | [7, 8] |
| x_TP_ | % | TP mass fraction of droplet | 0.896 | [7, 8] |
| $\theta_{NaCl}$ | - | Practical osmotic coefficient of NaCl | 3.75 | [7] |
| $\theta_{TP}$ | - | Practical osmotic coefficient of TP | 0.95 | [7] |
| g | m.sec^-2^ | Gravitational acceleration | 9.81 | - |
| ɳ | g.m^-1^.sec^-1^ | Air viscosity | 0.0185 | - |
| H | m | Settling Height | 1.5 | Distance from the source (patient’s mouth) to the ground |
| AI | m^3^.min^-1^ | Air Intake | 8.5 | The air intake was obtained from the AUBMC physical plant |
| V | m^3^ | Patient room volume | 70 | The volume was obtained from the AUBMC physical plant |

AUBMC: American University of Beirut Medical Center; TP: Total Particulate

Table S2: PM_10_ and PM_2.5_ data

| Date | PM_10_ (µg/m^3^) | | | PM_2.5_ (µg/m^3^) | | | Occupancy | T (C) | RH (%) |
| --- | --- | --- | --- | --- | --- | --- | --- | --- | --- |
|  | Average | Minimum | Maximum | Average | Minimum | Maximum |  |  |  |
| 12/27/2018 |  |  |  |  |  |  | 2 | 22.8 | 49.9 |
| 12/27/2018 | 15 | 13 | 16 | 11 | 11 | 11 | 2 | 23.8 | 45.4 |
| 12/27/2018 | 17 | 16 | 18 | 15 | 13 | 19 | 3 | 24.2 | 44.9 |
| 12/27/2018 | 14 | 13 | 16 | 13 | 13 | 13 | 2 | 24.3 | 45 |
| 12/28/2018 | 35 | 29 | 68 | 18 | 17 | 20 | 5 | 22.5 | 51.7 |
| 12/31/2018 | 18 | 18 | 19 | 16 | 16 | 17 | 3 | 22.9 | 53.2 |
| 12/31/2018 | 17 | 15 | 21 | 15 | 13 | 16 | 3 | 23.9 | 51.7 |
| 1/2/2019 | 41 | 35 | 48 | 33 | 31 | 35 | 3 | 22.2 | 45.2 |
| 1/2/2019 | 25 | 23 | 26 | 21 | 20 | 23 | 3 | 23.8 | 40.6 |
| 1/2/2019 | 32 | 31 | 33 | 29 | 28 | 30 | 3 | 24.5 | 40.7 |
| 1/2/2019 | 31 | 29 | 33 | 28 | 27 | 30 | 2 | 24.5 | 44.2 |
| 1/2/2019 | 46 | 42 | 49 | 39 | 37 | 40 | 5 | 25 | 38.7 |
| 1/4/2019 | 27 | 25 | 29 | 21 | 20 | 22 | 3 | 22.5 | 49 |
| 1/7/2019 | 9 | 8 | 10 | 7 | 7 | 7 | 3 | 21.8 | 47.9 |
| 1/9/2019 | 21 | 18 | 24 | 13 | 12 | 15 | 3 | 22.6 | 42.8 |
| 1/9/2019 | 19 | 17 | 22 | 15 | 13 | 18 | 3 | 24 | 40.8 |
| 1/9/2019 | 26 | 19 | 45 | 17 | 13 | 21 | 3 | 25 | 44.1 |
| 1/14/2019 |  |  |  |  |  |  | 3 | 22.7 | 53.1 |
| 1/16/2019 | 19 | 16 | 22 | 9 | 8 | 12 | 3 | 22.5 | 45.5 |
| 1/21/2019 | 59 | 55 | 64 | 47 | 45 | 48 | 2 | 22.7 | 48.7 |
| 1/21/2019 | 48 | 42 | 55 | 35 | 31 | 38 | 3 | 23.7 | 44.2 |
| 1/23/2019 | 59 | 53 | 72 | 45 | 43 | 46 | 2 | 21.1 | 45 |
| 1/23/2019 | 31 | 28 | 36 | 23 | 21 | 24 | 4 | 23.1 | 50.1 |
| 1/29/2019 | 43 | 30 | 54 | 26 | 24 | 29 | 2 | 20.6 | 59.3 |
| 1/29/2019 | 20 | 17 | 23 | 14 | 13 | 15 | 2 | 23.4 | 51 |
| 1/29/2019 | 12 | 11 | 15 | 10 | 9 | 11 | 3 | 25.4 | 48.7 |
| 1/30/2019 | 71 | 65 | 77 | 53 | 48 | 56 | 2 | 21.2 | 56.8 |
| 2/4/2019 | 53 | 48 | 56 | 50 | 30 | 70 | 2 | 22 | 47.2 |
| 2/8/2019 | 78 | 70 | 90 | 42 | 39 | 46 | 2 | 21.7 | 42.7 |
| 2/11/2019 | 43 | 23 | 60 | 21 | 17 | 38 | 3 | 21 | 59.9 |
| 2/11/2019 | 26 | 24 | 28 | 21 | 20 | 22 | 3 | 23.2 | 54.5 |
| 2/13/2019 | 25 | 22 | 30 | 18 | 17 | 20 | 2 | 21 | 51.3 |
| 2/27/2019 | 89 | 57 | 131 | 36 | 31 | 49 | 3 | 23.5 | 62.6 |

Table S3: Virus data

| Date | Number of Coughs | Distance | Flu Detection | Ct | Starting quantity /µL | Starting quantity /2 µL | Elution Volume (µL) | Extraction Volume (µL) | RNA Copies/m^3^ | Normalized RNA Copies /m^3^ |
| --- | --- | --- | --- | --- | --- | --- | --- | --- | --- | --- |
| 12/27/2018 | 0 | 1 | Negative | 0 |  |  | 40 | 500 | 0 | 0 |
|  | 1 | 0.5 | Negative | 0 |  |  | 40 | 500 | 0 | 0 |
| 12/27/2018 | 6 | 1 | Negative | 0 |  |  | 40 | 500 | 0 | 0 |
|  | 25 | 0.5 | Negative | 0 |  |  | 40 | 500 | 0 | 0 |
| 12/27/2018 | 23 | 1 | Negative | 0 |  |  | 40 | 500 | 0 | 0 |
|  | 20 | 0.5 | Negative | 0 |  |  | 40 | 500 | 0 | 0 |
| 12/27/2018 | 8 | 1 | Negative | 0 |  |  | 40 | 500 | 0 | 0 |
|  |  | 0.5 |  |  |  |  |  |  |  |  |
| 12/28/2018 | 18 | 1 | Positive | 35 | 11.3 | 5.6 | 40 | 500 | 1805 | 100.3 |
|  | 1 | 0.5 | Positive | 37 | 2.1 | 1.06 | 40 | 500 | 339.4 | 339.4 |
| 12/31/2018 | 7 | 1 | Negative | 0 |  |  | 40 | 500 | 0 | 0 |
|  | 5 | 0.5 | Negative | 0 |  |  | 40 | 500 | 0 | 0 |
| 12/31/2018 | 4 | 1 | Negative | 0 |  |  | 40 | 500 | 0 | 0 |
|  | 3 | 0.5 | Negative | 0 |  |  | 40 | 500 | 0 | 0 |
| 1/2/2019 | 0 | 1 | Negative | 0 |  |  | 40 | 500 | 0 | 0 |
|  | 0 | 0.5 | Negative | 0 |  |  | 40 | 500 | 0 | 0 |
| 1/2/2019 | 15 | 1 | Negative | 0 |  |  | 40 | 500 | 0 | 0 |
|  | 13 | 0.5 | Negative | 0 |  |  | 40 | 500 | 0 | 0 |
| 1/2/2019 | 5 | 1 | Negative | 0 |  |  | 40 | 500 | 0 | 0 |
|  | 1 | 0.5 | Negative | 0 |  |  | 40 | 500 | 0 | 0 |
| 1/2/2019 | 0 | 1 | Negative | 0 |  |  | 40 | 500 | 0 | 0 |
|  | 0 | 0.5 | Negative | 0 |  |  | 40 | 500 | 0 | 0 |
| 1/2/2019 | 1 | 1 | Positive | 37 | 2.5 | 1.2 | 40 | 500 | 393.1 | 393.1 |
|  | 0 | 0.5 | Negative | 0 |  |  | 40 | 500 | 0 | 0 |
| 1/4/2019 | 1 | 1 | Negative | 0 |  |  | 40 | 500 | 0 | 0 |
|  | 1 | 0.5 | Negative | 0 |  |  | 30 | 500 | 0 | 0 |
| 1/7/2019 | 7 | 1 | Positive | 37 | 2.4 | 1.2 | 30 | 500 | 290.6 | 41.5 |
|  | 4 | 0.5 | Negative | 0 |  |  | 30 | 500 | 0 | 0 |
| 1/9/2019 | 8 | 1 | Negative | 0 |  |  | 30 | 500 | 0 | 0 |
|  | 6 | 0.5 | Positive | 36 | 6.1 | 3 | 30 | 500 | 730.6 | 121.8 |
| 1/9/2019 | 5 | 1 | Negative | 0 |  |  | 30 | 500 | 0 | 0 |
|  | 4 | 0.5 | Positive | 36 | 3.1 | 1.5 | 30 | 500 | 367 | 92.5 |
| 1/9/2019 | 12 | 1 | Positive | 37 | 2.5 | 1.25 | 30 | 500 | 298.8 | 24.9 |
|  | 6 | 0.5 | Positive | 36 | 2.8 | 1.4 | 30 | 500 | 337.2 | 56.2 |
| 1/14/2019 | 0 | 1 | Negative | 0 |  |  | 30 | 500 | 0 | 0 |
|  | 0 | 0.5 | Negative | 0 |  |  | 30 | 500 | 0 | 0 |
| 1/16/2019 | 1 | 1 | Positive | 37 | 1.85 | 0.93 | 30 | 500 | 221.9 | 221.9 |
|  | 1 | 0.5 | Positive | 37 | 2.2 | 1.1 | 30 | 500 | 264.7 | 264.7 |
| 1/21/2019 | 9 | 1 | Positive | 37 | 2.6 | 1.3 | 30 | 500 | 308.2 | 34.2 |
|  | 8 | 0.5 | Negative | 0 |  |  | 30 | 500 | 0 | 0 |
| 1/21/2019 | 8 | 1 | Negative | 0 |  |  | 30 | 500 | 0 | 0 |
|  | 0 | 0.5 | Negative | 0 |  |  | 30 | 500 | 0 | 0 |
| 1/23/2019 | 30 | 1 | Positive | 37 | 1.9 | 0.94 | 30 | 500 | 224.8 | 7.5 |
|  | 26 | 0.5 | Positive | 37 | 2.5 | 1.3 | 30 | 500 | 302.5 | 11.6 |
| 1/23/2019 | 4 | 1 | Negative | 0 |  |  | 30 | 500 | 0 | 0 |
|  | 9 | 0.5 | Negative | 41 | 0.095 | 0.05 | 30 | 500 | 0 | 0 |
| 1/29/2019 | 11 | 1 | Positive | 36 | 6 | 3 | 30 | 500 | 724.2 | 65.8 |
|  | 4 | 0.5 | Negative | 0 |  |  | 30 | 500 | 0 | 0 |
| 1/29/2019 | 7 | 1 | Positive | 37 | 2.4 | 1.2 | 30 | 500 | 290.5 | 41.5 |
|  | 28 | 0.5 | Negative | 0 |  |  | 30 | 500 | 0 | 0 |
| 1/29/2019 | 1 | 1 | Positive | 33 | 48 | 24 | 30 | 500 | 5760 | 5760 |
|  | 0 | 0.5 | Negative | 0 |  |  | 30 | 500 | 0 | 0 |
| 1/30/2019 | 13 | 1 | Negative | 0 |  |  | 30 | 500 | 0 | 0 |
|  | 28 | 0.5 | Negative | 0 |  |  | 30 | 500 | 0 | 0 |
| 2/4/2019 | 0 | 1 | Negative | 0 |  |  | 30 | 500 | 0 | 0 |
|  | 0 | 0.5 | Negative | 0 |  |  | 30 | 500 | 0 | 0 |
| 2/8/2019 | 3 | 1 | Positive | 36 | 4.8 | 2.4 | 30 | 500 | 575.4 | 191.8 |
|  | 3 | 0.5 | Negative | 0 |  |  | 30 | 500 | 0 | 0 |
| 2/11/2019 | 8 | 1 | Negative | 0 |  |  | 30 | 500 | 0 | 0 |
|  | 10 | 0.5 | Negative | 0 |  |  | 30 | 500 | 0 | 0 |
| 2/11/2019 | 2 | 1 | Negative | 0 |  |  | 30 | 500 | 0 | 0 |
|  | 0 | 0.5 | Negative | 0 |  |  | 30 | 500 | 0 | 0 |
| 2/13/2019 | 0 | 1 | Negative | 0 |  |  | 30 | 500 | 0 | 0 |
|  | 0 | 0.5 | Negative | 0 |  |  | 30 | 500 | 0 | 0 |
| 2/27/2019 | 9 | 1 | Positive | 34 | 26.9 | 13.4 | 30 | 500 | 3225.6 | 358.4 |
|  | 10 | 0.5 | Positive | 36 | 3.7 | 1.8 | 30 | 500 | 437.5 | 43.8 |

Table S4: droplets diameter ranges emitted during coughing
[9]

| Average Diameter (µm) | Range (µm) | Droplet Percentage (%) |
| --- | --- | --- |
| 1.5 | 1-2 | 0 |
| 3 | 2-4 | 0 |
| 6 | 4-8 | 0 |
| 12 | 8-16 | 0 |
| 20 | 16-24 | 0.3 |
| 28 | 24-32 | 0 |
| 36 | 32-40 | 0.5 |
| 45 | 40-50 | 6.2 |
| 62.5 | 50-75 | 30.8 |
| 87.5 | 75-100 | 23.4 |
| 112.5 | 100-125 | 15.0 |
| 137.5 | 125-150 | 5.9 |
| 175 | 150-200 | 7.4 |
| 225 | 200-250 | 3.4 |
| 375 | 250-500 | 4.5 |
| 750 | 500-1000 | 2.4 |
| 1500 | 1000-2000 | 0.2 |

**REFERENCES**

1. Gupta, J.K., Lin, C.-H. and Chen, Q., 2009. Flow Dynamics and Characterization of a Cough. Indoor Air, 19: 517-525.
2. Kwon, S.B., Park, J., Jang, J., Cho, Y., Park, D.S., Kim, C., Bae, G.N. and Jang, A., 2012. Study on the initial velocity distribution of exhaled air from coughing and speaking. Chemosphere, 87(11): 1260-4.
3. Tang, J.W., Nicolle, A., Pantelic, J., Koh, G.C., Wang, L.D., Amin, M., Klettner, C.A., Cheong, D.K.W., Sekhar, C. and Tham, K.W., 2012. Airflow Dynamics of Coughing in Healthy Human Volunteers by Shadowgraph Imaging: An Aid to Aerosol Infection Control. PLoS one, 7(4).
4. Chamseddine, A., 2018. Determinants of Indoor Air Quality in Hospitals: Impact of ventilation systems with Indoor-Outdoor correlations and health implications. PhD Dissertation, Department of Civil and Environmental Engineeing, American University of Beirut, Lebanon.
5. Lindsley, W.G., Blachere, F.M., Thewlis, R.E., Vishnu, A., Davis, K.A., Cao, G., Palmer, J.E., Clark, K.E., Fisher, M.A., Khakoo, R. and Beezhold, D.H., 2010. Measurements of airborne influenza virus in aerosol particles from human coughs. PLoS One, 5(11): e15100.
6. Mikhailov, E., Vlasenko, S., Niessner, R. and Pöschl, U., 2004. Interaction of aerosol particles composed of protein and saltswith water vapor: hygroscopic growth and microstructural rearrangement. Atmospheric Chemistry and Physics, 4(2): 323-350.
7. Yang, W. and Marr, L.C., 2011. Dynamics of airborne influenza A viruses indoors and dependence on humidity. PLoS One, 6(6): e21481.
8. Nicas, M., Nazaroff, W.W. and Hubbard, A., 2005. Toward understanding the risk of secondary airborne infection: emission of respirable pathogens. J Occup Environ Hyg, 2(3): 143-54.
9. Xie, X., Li, Y., Sun, H. and Liu, L., 2009. Exhaled droplets due to talking and coughing. J R Soc Interface, 6 Suppl 6: S703-14.
